# Synthesis of Hierarchically Porous Bioactive Glass and Its Mineralization Activity

**DOI:** 10.3390/molecules28052224

**Published:** 2023-02-27

**Authors:** Jiawei Liu, Guo Du, Hongda Yu, Xueyin Zhang, Tiehong Chen

**Affiliations:** Interdisciplinary Science Center, Key Laboratory of Advanced Energy Materials Chemistry (MOE), Institute of New Catalytic Materials Science, School of Materials Science and Engineering, Smart Sensing, Nankai University, Tianjin 300350, China

**Keywords:** bioactive glass, hierarchically porous structure, mesoporous material, hydroxyapatite, self-assembly

## Abstract

Mesoporous bioactive glass is a promising biomaterial for bone tissue engineering due to its good biocompatibility and bioactivity. In this work, we synthesized a hierarchically porous bioactive glass (HPBG) using polyelectrolyte-surfactant mesomorphous complex as template. Through the interaction with silicate oligomers, calcium and phosphorus sources were successfully introduced into the synthesis of hierarchically porous silica, and HPBG with ordered mesoporous and nanoporous structures was obtained. The morphology, pore structure and particle size of HPBG can be controlled by adding block copolymer as co-template or adjusting the synthesis parameters. The ability to induce hydroxyapatite deposition in simulated body fluids (SBF) demonstrated the good in vitro bioactivity of HPBG. Overall, this work provides a general method for the synthesis of hierarchically porous bioactive glasses.

## 1. Introduction

Silicate-based bioactive materials have attracted wide attention because of their applications in bone tissue engineering and drug delivery [1,2,3,4]. Bioactive glass was proposed by Hench et al. in 1971 [5], with SiO_2_, CaO and P_2_O_5_ as the main components, having good bioactivity and biocompatibility. After being implanted into the organism, bioactive glass can induce the formation of hydroxyapatite layer on its surface and combine with the bone in vivo, which makes it widely used in clinical practice for bone repair [6,7,8,9]. With large specific surface area and pore volume, hierarchically porous silica can be used as scaffold for hard tissues in organisms, and it can also be loaded with osteogenic agents to promote the formation of new bone [10,11,12]. Since the development of mesoporous bioactive glass by Zhao et al. [13] in 2004, porous bioactive glass related to bone regeneration has also attracted researchers’ wide attention [14,15]. However, the unitary pore size of the mesopore limits the practical application of bioactive glass to some extent [16]. Studies have shown that biomaterials used in bone tissue engineering need pore structures of different scales to play different roles [17]. The existence of hierarchically porous structure can ensure abundant blood supply, adequate gas exchange and effective nutrient transport in applications [18,19]. Yun et al. [20] synthesized a hierarchically microporous–mesoporous bioactive glass scaffold by sol-gel method using double polymers as template, and proved that the scaffold has good in vitro bone-forming bioactivity by experiments. The microporous–mesoporous structure of the scaffold can enhance tissue oxygenation and has the function of loading multiple drugs for controlled release. Han et al. [21] synthesized a mesoporous–macroporous bioactive glass and carried out drug loading and release tests using ibuprofen as a model drug. The results showed that the bioactive glass has high drug loading and drug release ability. In addition, due to the unique hierarchically porous structure, the material has excellent biomineralization activity and biocompatibility, which offers it unique properties in the field of bone regeneration and drug delivery. Li et al. [22] prepared a hierarchically porous bioactive glass scaffold, using P123 and polyurethane (PUF) as co-templates. In the synthesis, PUF functioned as a positive hard template for interconnected macroporous structures, and P123 as a soft template for the uniform mesopores. The hierarchically porous structure exhibits both advantages of different pore sizes, enhancing its potential applications in tissue engineering and drug storage. Shih et al. [23] prepared bioactive glass powders using either a polyurethane foam sponge-F127 co-template (marked as SCP-CoT) or only a polyurethane foam sponge macrotemplate (marked as SCP-T), and used them to evaluate the gentamicin encapsulation of the hierarchically bioactive glass. The hierarchically porous bioactive glass SCP-CoT has approximately 3-times larger specific surface area and 5-times larger pore volume compared to that of macroporous SCP-T sample, which results in a significant enhancement of the encapsulation capacity of gentamicin, from 5.4% (SCP-T) to 29.4% (SCP-CoT). The properties of bioactive glass are closely related to its composition, porosity, specific surface area and particle size [24,25,26,27]. Increasing the specific surface area and pore volume of bioactive glass can greatly accelerate the dynamic deposition of hydroxyapatite, thus improve its bioactivity [28,29]. In addition, the mesoporous structure in bioactive glass has efficient immobilization ability for some biomolecules, such as proteins and drug molecules, which is helpful for bone repair and regeneration [30,31,32].

Sol-gel method is one of the most commonly used methods for the synthesis of mesoporous bioactive glass [33,34]. Compared with the high temperature melting process, this method can obtain mesoporous bioactive glass with high porosity at low temperature [35,36,37]. A variety of surfactants can be added to the sol-gel synthesis system to introduce mesoporous structures into bioactive glass [38,39]. With the mesomorphous complex formed by polyacrylic acid (PAA) and surfactant CTAB as template, our group had firstly prepared NKM-5, a hierarchically porous silica material with ordered mesoporous structure [40]. Inspired by the previous works, herein we report the synthesis of a hierarchically porous bioactive glass (HPBG) by adding phosphorus source (triethyl phosphate) and calcium source (calcium nitrate) to the synthesis system of hierarchically porous silica, using polyelectrolyte-surfactant mesomorphous complex as template. Through the interaction with silicate oligomers, calcium and phosphorus sources can be successfully introduced into the hierarchically porous silica to obtain HPBG. Moreover, the morphology, pore structure and particle size of the synthesized materials can be controlled by adding block copolymer as co-template or adjusting the synthesis parameters, and hierarchically porous bioactive glasses with rod-like and nano-particle shapes were prepared. This is the first time that a synthesis of a bioactive glass using an ionic polymer-surfactant complex as a co-template is reported.

## 2. Results and Discussion

### 2.1. Synthesis of Hierarchically Porous Bioactive Glass

**Figure 1:**
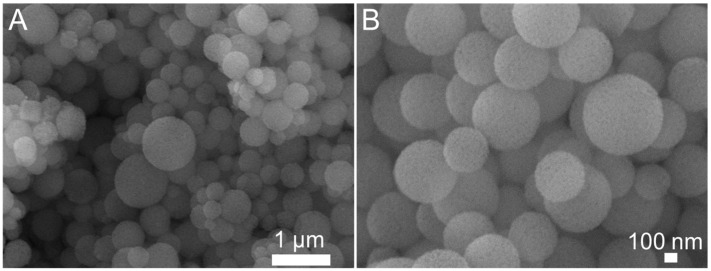
(**A**,**B**) SEM images of calcined samples HPBG with different magnification.

**Figure 2:**
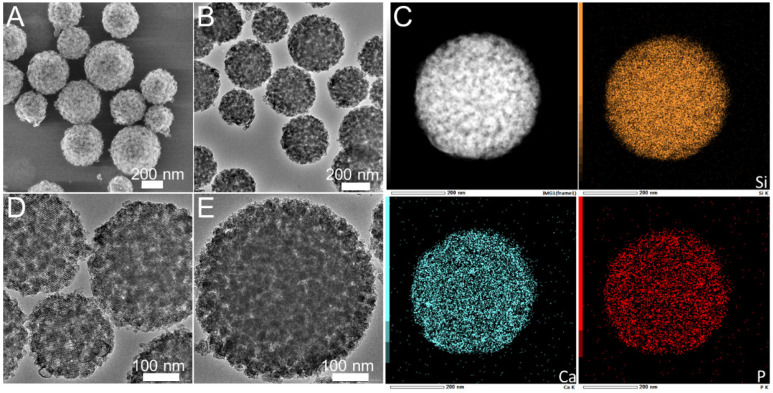
(**A**,**B**,**D**,**E**) TEM images; (**C**) EDS-mapping of calcined samples HPBG.

**Figure 3:**
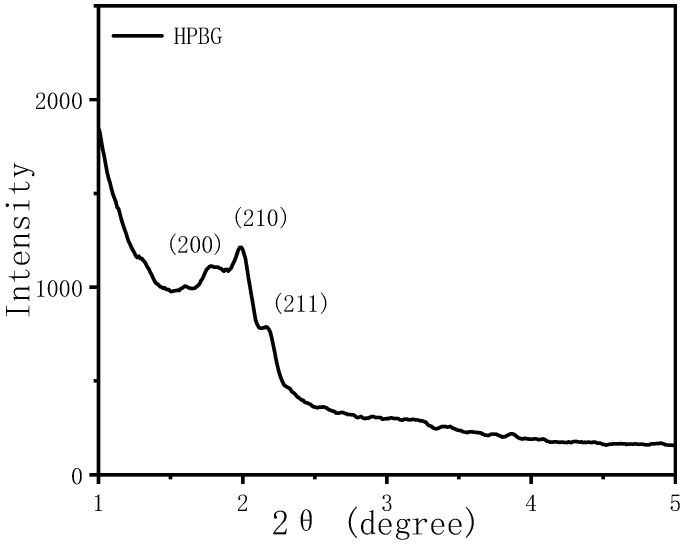
Low-angle XRD pattern of calcined samples HPBG.

The morphology and pore structure of the bioactive glass HPBG were analyzed by SEM and TEM. As shown in Figure 1, using the polyelectrolyte-surfactant mesomorphous complex as template, the prepared HPBG has regular spherical morphology with particle size of 500~800 nm and a large number of groove-like structures on the surface. The spherical morphology of the sample can also be clearly observed by TEM (Figure 2B). The different contrast inside the particles indicates the existence of nanoporous structure with large pore size in HPBG. In addition, a substantial amount of mesopores in HPBG particles can be observed by high-magnification transmission electron microscopy (Figure 2D), and the mesoporous structure is highly ordered and oriented. The existence of the secondary mesopores does not destroy the directional arrangement of the mesoporous structure, and the whole particle exhibits the properties of a single crystal. According to the EDS-mapping (Figure 2C), the three elements Si, Ca and P in HPBG are uniformly distributed, and the contents are 78% atom, 13% atom and 9% atom, respectively. The small angle XRD (Figure 3) shows three distinct diffraction peaks in the range of 2θ = 1.0~3.5°, which are indexed to (200), (210) and (211) characteristic diffractions of the cubic *Pm-3n* mesostructure symmetry. This indicates an ordered mesoscopic structure in the HPBG sample, which is consistent with the results observed by TEM.

**Figure 4:**
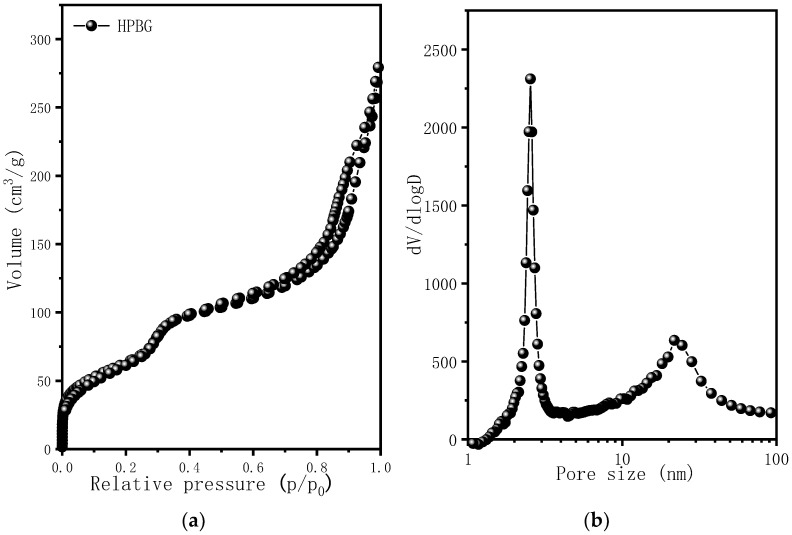
(**a**) Nitrogen adsorption–desorption isotherms; (**b**) pore size distribution curve of calcined samples HPBG.

Figure 4 is the nitrogen adsorption–desorption isotherm and the corresponding pore size distribution curve of the hierarchically porous bioactive glass HPBG. The isotherm is a typical type IV adsorption–desorption isotherm with two distinct adsorption steps at the relative pressure of 0.30~0.50 and 0.7~0.99, indicating that the sample has mesoporous structure. The first adsorption step corresponds to the narrow peak at 3 nm in the pore size distribution curve, which is the ordered mesoporous structure in the material (corresponding to the ordered mesoporous structure shown in Figure 2D). The second adsorption step corresponds to the secondary mesopores in the material, which can be clearly observed by TEM and relates to the broad peak centered at 20 nm in the pore size distribution curve. The synthesized porous bioactive glass has large specific surface area and pore volume, which are 623 m^2^g^−1^ and 0.87 cm^3^g^−1^, respectively.

From the above characterization and analysis, a conclusion can be drawn that when adding phosphorus source (triethyl phosphate) and calcium source (calcium nitrate) into the synthesis system, Ca and P can be introduced into silica material with the help of silicate oligomer. Compared with the traditional mesoporous bioactive glass, the hierarchically porous bioactive glass prepared by the proposed method has not only ordered mesoporous structure, but also larger nanoporous structure. It has high specific surface area (623 m^2^g^−1^) and pore volume (0.87 cm^3^g^−1^).

### 2.2. Morphology Control of Hierarchically Porous Bioactive Glass

**Figure 5:**
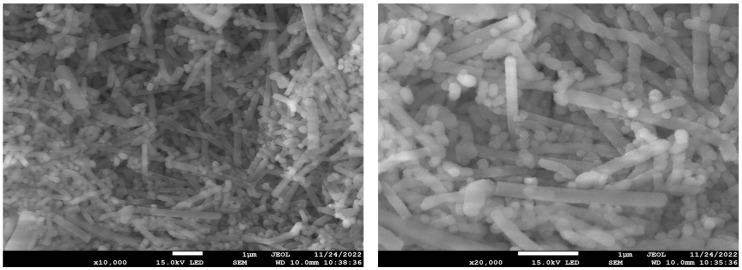
SEM image of R-HPBG.

**Figure 6:**
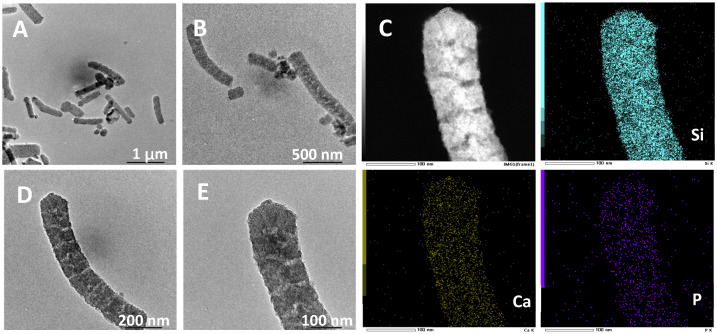
(**A**,**B**,**D**,**E**) TEM images; (**C**) EDS-mapping of calcined samples R-HPBG.

**Figure 7:**
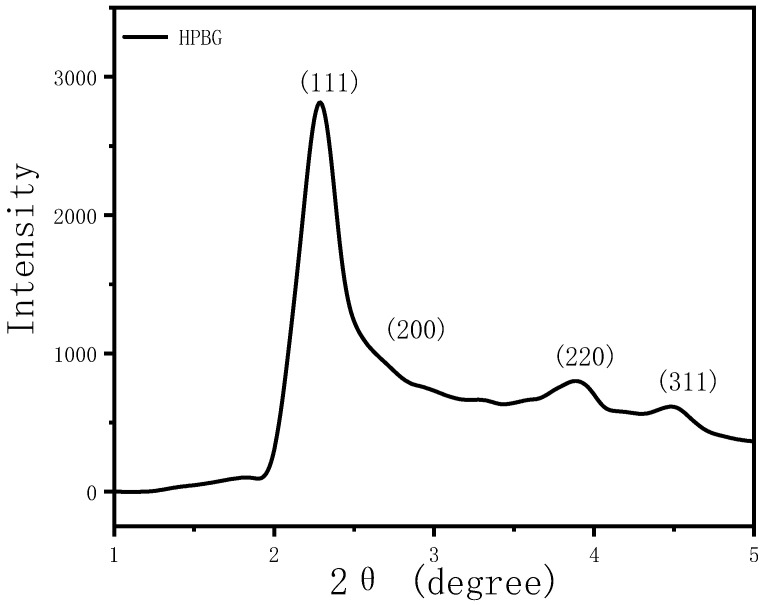
Low-angle XRD patterns of calcined samples R-HPBG.

**Figure 8:**
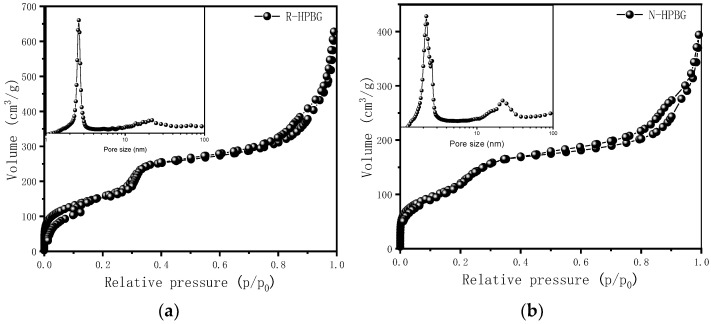
Nitrogen adsorption–desorption isotherms and pore size distribution curves of (**a**) R-HPBG and (**b**) N-HPBG.

In the preparation of hierarchically porous silica, we have found that the addition of block copolymer P123 (PEO_20_PPO_70_PEO_20_) can realize the morphology modulation of the material [41]. Inspired by this, we introduced P123 into the synthesis system. Bioactive glass nanorods R-HPBG with ordered mesopores and secondary mesopores were synthesized by using cetyltrimethylammonium bromide (CTAB) as template and polyacrylic acid (PAA) and triblock copolymer P123 as co-template. SEM images show the rod-shaped morphology with a diameter of 300–500 nm of the sample (Figure 5). The different contrast in the TEM image (Figure 6) indicates the presence of secondary mesopores within the nanorods. In addition, an ordered mesoporous structure can be observed through the high-magnification transmission electron microscope. Despite the existence of secondary mesopores, the mesopores still maintain a good long-range order, which indicates that the existence of secondary mesopores does not destroy the orientation of the mesoporous structure, and the entire particle still maintains the properties of a single crystal. According to EDS-mapping (Figure 6C), similar to the HPBG sample, the distribution of Si, Ca and P in the nanorods are also uniform, and the contents are 83% atom, 9% atom and 8% atom, respectively. The small angle XRD (Figure 7) of the HPBG sample shows four distinct diffraction peaks indexed to (111), (200), (220) and (311) characteristic diffractions of the cubic *Fm-3m* mesostructure symmetry. The nitrogen adsorption–desorption curve (Figure 8a) of the sample shows a typical IV isotherm with three different adsorption steps at relative pressures of 0.3~0.5, 0.7~0.95 and 0.95~0.99. The first step corresponds to the relatively narrow peak in the pore size distribution curve at 2.2 nm, which is the cage-like channel of three-dimensional cubic *Fm-3m* mesoscopic structure. The second step corresponds to the relatively wide peak in the pore size distribution curve, which is the secondary mesopores observed in SEM and TEM images. The third step is the interstitial pores formed by the stacking of nanorods. The structural information of the sample is listed in Table 1.

The above results indicate that the morphology and mesoscopic structure of the hierarchically porous bioactive glass can be controlled by adding block copolymer P123 as co-template.

### 2.3. Particle Size Control of Hierarchically Porous Bioactive Glass

**Figure 9:**
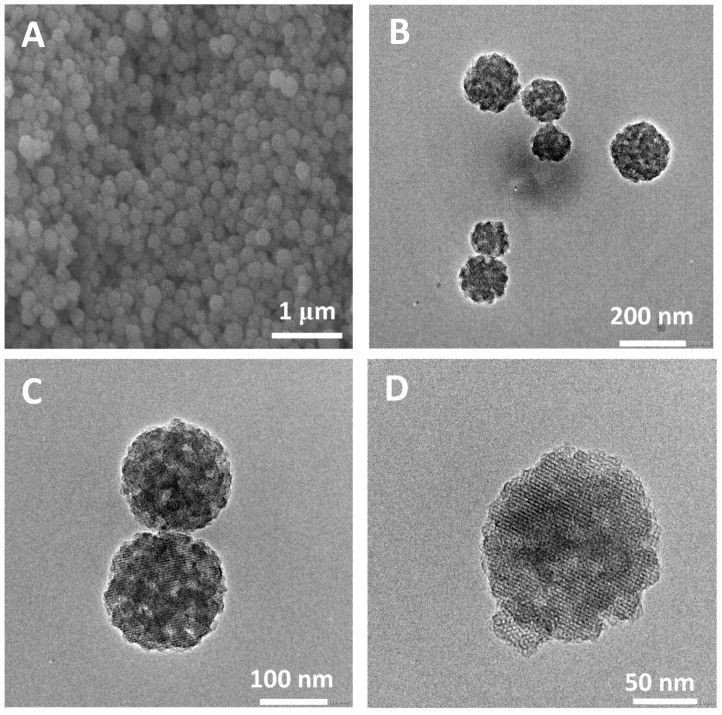
(**A**) SEM; (**B**–**D**) TEM images of N-HPBG.

**Figure 10:**
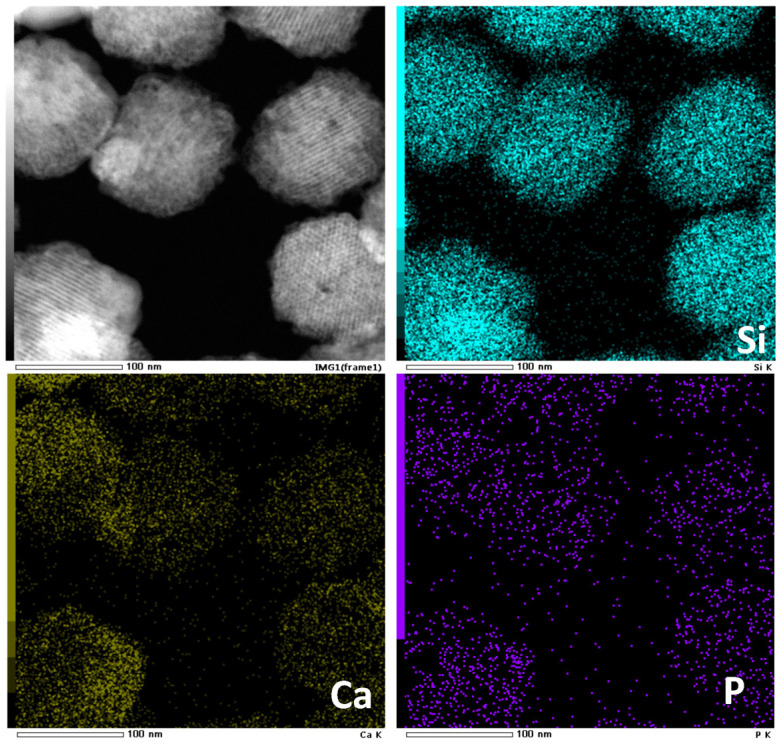
EDS-mapping of N-HPBG.

Nanoparticles with regular morphology and uniform size have many unique physical and chemical properties, such as quantum size effect, high surface area to volume ratio and surface effect, which make nanoparticles widely used in biomedicine [42,43,44]. On the basis of the successful synthesis of HPBG microspheres, adjusting the synthesis parameters, such as the amount of surfactant and polyelectrolyte, can realize the regulation of the particle size and achieve the preparation of hierarchically porous bioactive glass nanoparticles (N-HPBG). Figure 9 shows the SEM and TEM images of the synthesized bioactive glass nanoparticles, from which it can be clearly observed that the sample has a regular spherical morphology with the particle size of about 100~200 nm. Further analysis of the morphology and internal pore structure by TEM can also obtain the regular spherical morphology of the sample, which is consistent with the results by SEM. In addition, different contrasts inside the particles can be observed by HR-TEM, indicating that there are secondary mesopores with larger sizes in the particles. The uniform distribution of Si, Ca and P can be confirmed by EDS-mapping (Figure 10), and the contents of Si, Ca and P are 82% atom, 12% atom and 6% atom, respectively.

**Figure 11:**
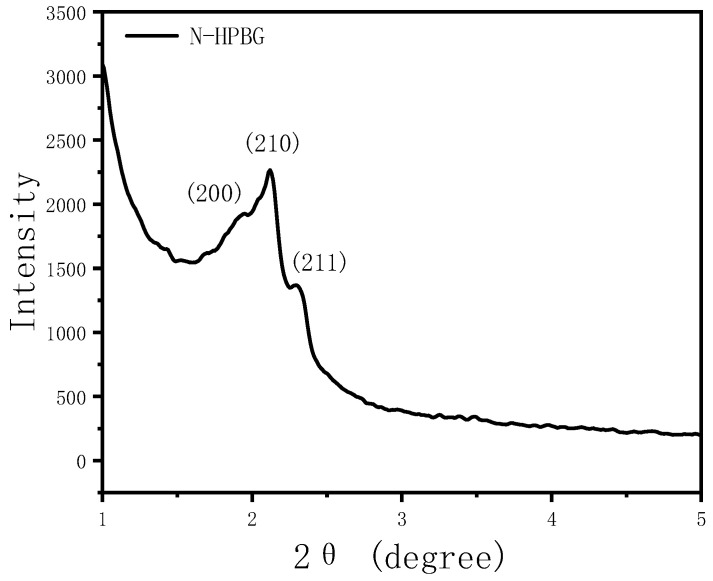
Low-angle XRD pattern of N-HPBG.

The small angle XRD (Figure 11) of N-HPBG shows three distinct diffraction peaks indexed to (200), (210) and (211) characteristic diffractions of the cubic *Pm-3n* mesostructure symmetry, indicating the existence of ordered mesoporous structure. The isotherm of this sample shows a typical type IV adsorption–desorption isotherm (Figure 8b), and the adsorption step at the relative pressure of 0.30–0.50 is formed by the ordered mesoporous structure, which corresponds to the peak of about 2.2 nm in the pore size distribution curve. The adsorption step at the relative pressure of 0.75–0.95 indicates the existence of secondary mesopores, which can be clearly observed by TEM and which corresponds to the wide peak in the range of 14~30 nm in the pore size distribution curve. The adsorption step at the relative pressure of 0.95–0.99 is the interstitial pores formed by the accumulation of nanoparticles. The structural parameters of the samples are listed in Table 1, in which the specific surface area and pore volume of hierarchically porous bioactive glass nanoparticles are 605 m^2^g^−1^ and 0.52 cm^3^g^−1^, respectively.

Combining the TEM images, small-angle XRD pattern and nitrogen adsorption data, it can be concluded that hierarchically porous bioactive glass nanoparticles with ordered mesoporous structure and secondary mesopores can be prepared by adjusting the synthesis parameters in the synthesis system, using PAA-CTAB mesomorphous complex as template.

### 2.4. In Vitro Bioactivity Test of HPBG

**Figure 12:**
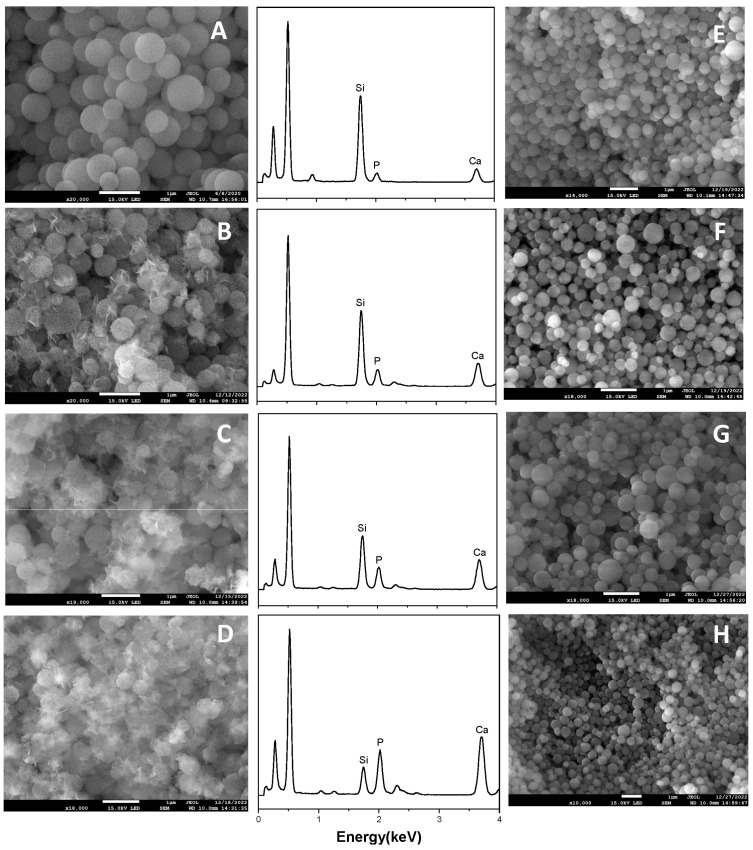
(**A–D**) SEM images and EDS curves of HPBG after soaking in SBF for 0, 24, 48, 72 h; (**E–H**) SEM images of NKM-5 after soaking in SBF for 0, 24, 48, 72 h.

**Figure 13:**
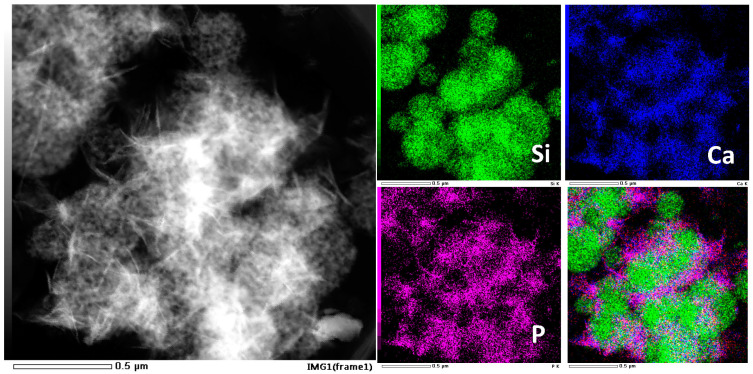
EDS-mapping of HPBG after soaking in SBF for 72 h.

**Figure 14:**
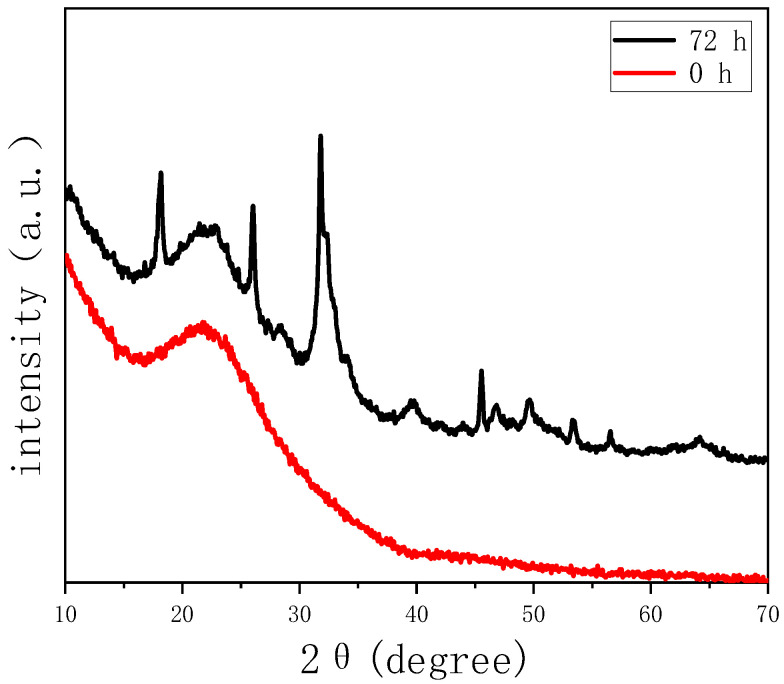
Wide-angle XRD patterns of HPBG before and after soaking in SBF for 72 h.

The capacity of growing hydroxyapatite on the surface of a biomaterial when soaked in simulated body fluid reflects its ability of bonding to the human bone tissue in vivo, which is a significant descriptor of bioactivity. Therefore, we test the in vitro bioactivity of HPBG (compared with hierarchically porous silica microspheres NKM-5) in SBF. SEM images of the samples after soaking in SBF for different times (Figure 12) showed that needle-like crystal aggregates appeared on the sample surface after 24 h, and the surface was almost completely covered by crystals after 72 h. In contrast, the deposition rate of hydroxyapatite on the surface of NKM-5 is much slower, and no crystals can be observed after 72 h. EDS images showed that after soaking in SBF, the content of Ca and P on the surface of HPBG particles increased, while the content of Si decreased. EDS-mapping (Figure 13) shows the central silicon-rich HPBG particle and the surrounding Ca and P region more clearly. The XRD pattern of the sample soaked for 72 h (Figure 14) confirmed that the needle-like crystals on the surface can be attributed to the hydroxyapatite phase. The above results indicate that HPBG particles can rapidly induce the deposition of hydroxyapatite in SBF and have good bioactivity.

## 3. Materials and Methods

### 3.1. Materials

Cetyltrimethylammonium bromide (CTAB), tetraethyl orthosilicate (TEOS), ammonia (25–28% aqueous solution) and triethyl phosphate (99% RG) were purchased from Shanghai Aladdin Biochemical Technology Co., Ltd.(China) Polyacrylic acid (25 wt%, MW = 240,000) was purchased from Alfa Aesar, P123 (Mn = ~5800, EO_20_PO_70_EO_20_) was purchased from Sigma-Aldrich. Calcium nitrate tetrahydrate (Ca(NO_3_)_2_·4H_2_O) was purchased from Tianjin Jiangtian Chemical Technology Co., Ltd.(China) All materials were used as received without further purification.

### 3.2. Synthesis of Hierarchically Porous Bioactive Glass

The typical process of synthesizing hierarchically porous bioactive glass using polyacrylic acid (PAA) and cationic surfactant (CTAB) mesomorphous complex as template is as follows. An amount of 0.55 g CTAB was fully dissolved in 25 mL of deionized water. The solution was added with 4.0 g PAA (25 wt% aqueous solution) and stirring was maintained for 30 min until the solution was clear. Then, 4.0 g of ammonia (25~28 wt% aqueous solution) was added under stirring. Immediately, the solution became a milky emulsion. After stirring for 30 min, 2.08 g tetraethyl orthosilicate was added to the emulsion, which further reacted for 30 min until a white emulsion was obtained. The emulsion was then centrifuged to obtain a white product which was subsequently redispersed in 30 mL deionized water. An amount of 1.8 g calcium nitrate tetrahydrate and 0.9 g triethyl phosphate were added to the solution before stirring for another 20 min. After heating at 80 °C for 24 h, the product was centrifuged and washed with deionized water. Then, the product was calcinated at 600 °C to remove the organic template and obtain the hierarchically porous bioactive glass, which is named HPBG.

The synthesis process of rod-shaped hierarchically porous bioactive glass (R-HPBG) was similar to that of HPBG, except that P123 was added to the synthesis system for shape adjustment. The specific process is that after PAA and CTAB were fully dissolved, 4.0 g of P123 (5 wt% aqueous solution) was added and the solution was stirred for 20 min. The rest of the synthesis process was the same as that of HPBG.

The synthesis process of bioactive glass nanoparticles (N-HPBG) was similar to that of HPBG, except that the amount of template and silicon source was adjusted. The synthesis was carried out in a dilute solution so as to realize the regulation of the particle size. The specific amount of template and silicon source was CTAB 0.28 g, PAA 2.25 g, TEOS 1.04 g, and the other synthesis process was the same as that of HPBG.

### 3.3. Characterization of Hierarchically Porous Bioactive Glass

The XRD spectrum of HPBG powder was obtained by Rigaku Smart Lab 3kW diffractometer, with Cu K_α_ radiation at 40 kV and 100 mA. Small angle X-ray scattering (SAXS) patterns were taken on Bruker NanoSTAR. The morphology and microstructure were characterized by field emission scanning electron microscope SEM (JEOL, JSM-7800F) with operating voltage at 15 kV, and transmission electron microscope TEM (Philips Tecnai F20) equipped with X-ray energy spectrometer (EDS) with operating voltage at 200 kV. All the samples characterized by TEM were first dispersed in ethanol and then dripped onto the carbon film. The specific surface area and pore volume were measured by nitrogen adsorption analyzer (BELSORP-mini) at 77 K. Samples were degassed at 350 °C under nitrogen flow for at least 4 h prior to testing. The specific surface area and total pore volume of the materials were calculated by the BET (Brunauer–Emmett–Teller) method, and the pore size distribution was calculated by the BJH (Barrett–Joyner–Halenda) method based on the adsorption data.

### 3.4. In Vitro Bioactivity Test of HPBG

The in vitro bioactivity test of HPBG was carried out in simulated body fluid (SBF). This method was proposed by Kokubo et al., in which the ionic composition of SBF is similar to that of human plasma [45]. A total of 100 mg HPBG was soaked in 100 mL SBF and placed in a constant temperature environment of 37 °C. Each sample was put in at the same time and incubated for 0 to 72 h, respectively. The samples were subsequently filtered, washed and dried before further characterization, and the reaction time formed a time sequence. The deposition of hydroxyapatite on the material surface was investigated by SEM, TEM and XRD.

## 4. Conclusions

In this work, we have demonstrated a general method for synthesizing hierarchically porous bioactive glass (HPBG). As a conclusion, bioactive glasses with hierarchically porous structure were prepared by using polyelectrolyte-surfactant mesomorphous complex as template. Characterizations show that the synthesized HPBG has both ordered mesoporous structure of *Pm-3n* phase and secondary mesopores. The in vitro bioactivity of HPBG particles was tested in simulated body fluid (SBF). Under the induction of bioactive glass, a hydroxyapatite layer was formed on the surface, indicating that the HPBG has good bioactivity and biocompatibility. With the unique hierarchically porous structure and outstanding bioactivity, HPBG can be regarded as a potential material for bone tissue engineering. Moreover, bioactive glasses with different morphologies can be prepared by adjusting synthesizing parameters, which will be beneficial to meet the different requirements of practical applications. Overall, the unique hierarchically porous structure and outstanding bioactivity of the HPBG provide a potential material for bone tissue engineering.

## Figures and Tables

**Table 1:** Structural parameters of the calcined HPBG, R-HPBG and N-HPBG samples.

Sample	BET Surface Area/m^2^g^−1^	Mesopore Size/nm	Secondary Mesopores Size/nm	Total Volume ^a^/cm^3^g^−1^
HPBG	623	2.9	24	0.87
R-HPBG	693	2.6	21.8	0.98
N-HPBG	605	2.2	21.8	0.60

^a^ Calculated from *p*/*p*_0_ = 0.98 by the BET method.

## Data Availability

Not applicable.
